# A Cluster Randomized Controlled Trial Feasibility Study of a WhatsApp-Delivered Intervention to Promote Healthy Eating Habits in Male Firefighters

**DOI:** 10.3390/ijerph18126633

**Published:** 2021-06-20

**Authors:** Winnie Wing Man Ng, Anthony Siu Wo Wong, Kin Cheung

**Affiliations:** 1 Division of Science, Engineering and Health Studies, College of Professional and Continuing Education, No. 8 Hung Lok Road, Hung Hom, Hong Kong, China; 2 Centre for Gerontological Nursing, School of Nursing, The Hong Kong Polytechnic University, Hung Hom, Hong Kong, China; anthonysw.wong@connect.polyu.hk; 3 School of Nursing, The Hong Kong Polytechnic University, Hung Hom, Hong Kong, China

**Keywords:** fruit and vegetables consumption, cluster randomized control trial, health promotion, healthy eating habits, firefighters, feasibility study

## Abstract

This cluster randomized controlled trial (CRCT)-designed study aimed to explore the feasibility of a promotion pamphlet and/or WhatsApp as a suitable mode of delivery to promote healthy eating habits with fruit and vegetables (F&V) among firefighters. Convenience and snowball sampling methods were used. Forty-five firefighters from 23 fire stations were recruited and they all received the printed pamphlet, while the intervention group participants (*n* = 20) received additional teaching material through WhatsApp every two weeks for eight weeks. Feasibility outcomes included retention, practicality, and implementation. The participants reported high levels of satisfaction with the intervention. There were significant improvements in the mean numbers of days consuming F&V (*p =* 0.002; *p =* 0.031) in the intervention group, and for fruit consumption (*p =* 0.033) in the control group between the baseline (T_0_) and 3 months after completion of intervention (T_1_). High levels of participants’ satisfaction with the intervention revealed that a full-scale CRCT of the WhatsApp-delivered intervention promoting healthy eating could be feasible, especially as a means of increasing the numbers of days they consumed F&V and the numbers of servings of these consumed per day.

## 1. Introduction

Evidence has shown that fruit and vegetables (F&V) intake can reduce the risk of non-communicable diseases (NCDs) such as obesity and cardiovascular diseases (CVDs) [1,2,3]. Research has found that obesity or being overweight is common among firefighters in the United States, the United Kingdom, and Hong Kong (HK) [4,5,6]. In fact, being obese or overweight increases one’s risk of CVDs [7,8]. Among firefighters, CVDs is the leading cause of their on-duty deaths in the United States (US), with sudden cardiac death (SCD) contributing to about 45% of on-duty deaths [9,10,11]. Firefighters’ long shiftwork pattern could lead to desynchronized circadian rhythm, which could affect their hunger and satiety cues. This might induce unhealthy eating habits, such as overeating [12,13]. In general, inadequate intake of F&V is one of the major unhealthy eating habits [14]. In Western countries, about 28% of the US firefighters (*n* = 168) [15] and 75% of Californian firefighters (*n* = 268) [16] consumed less than the World Health Organization’s recommended daily five servings of F&V. A similar daily F&V intake pattern was also found in Eastern contexts. A study in HK found that over 93% of firefighters (*n* = 682) consumed less than two servings of fruit and more than 94% of them (*n* = 710) had less than two servings of vegetables per day [17]. The health of firefighters is of paramount importance, as their work is to protect the public. Furthermore, evidence has shown the protective effects of F&V intake on CVDs [18]. Dietary fiber could also provide different health benefits such as satiety [19]. Therefore, there is a need to increase their F&V consumption to reduce the risk of SCD.

Over the years, various face-to-face delivery methods, including seminars, educational videotapes, and pamphlets, were found to be effective ways to promote healthy eating habits [20]. However, these methods are not applicable to firefighters’ working environments due to their unique, special roster duty patterns and unpredictable working nature. For instance, US firefighters work on two consecutive 24 shifts and are then off for 96 h [21]; while United Kingdom firefighters work on a 2-2-4 shift pattern of 2 days, 2 nights and then 4 days off [22]. In HK [23] and South Korea [24], firefighters work “24 h on, 48 h off”. Furthermore, they can be called out at any time to extinguish fires or for other emergencies. Thus, a flexible and user-friendly method of promoting healthy eating should be adopted. Social networking platforms (SNP) may be a useful way to deliver health promotion programs [25]. For example, in 2017, around 1.5 billion people were using WhatsApp as their usual messaging service globally [26]. WhatsApp is a mobile instant messaging application that can deliver various formats of messages, including texts, images, audio, or videos, at any time, even if the users are offline or out of network coverage. Even when their devices are switched off when messages are sent, the users can still retrieve these messages when they turn on the device and open WhatsApp [27,28]; hence WhatsApp could be a tool to deliver health messages. Several studies have shown that healthy eating promotion programs delivered through WhatsApp can increase F&V consumption across different populations [29,30,31], but to date there is no evidence of its efficacy for special working groups such as firefighters.

The review of literature indicated that the application of theory to interventions can enhance changes in health behaviors [32]. The transtheoretical model (TTM) has been described as a credible theory for motivating changes in eating behaviors in various populations [25], including firefighters [33,34] in the US. However, the TTM-based health promotion program studies for firefighters in Eastern cultures have been limited. The TTM consists of five distinct stages of readiness for behavioral change: precontemplation, contemplation, preparation, action, and maintenance [35], with cognitive, motivational, and behavioral aspects for modifying lifestyle habits [36,37]. A systematic review found that TTM-based promotion of healthy eating could increase consumption of F&V [38]. However, it is unknown whether a TTM-based health promotion program could be as applicable to firefighters in Eastern countries as to their Western counterparts, due to the differences in eating cultures and working systems. Therefore, the purpose of this study was to investigate the feasibility and the potential effects of a WhatsApp-delivered intervention on firefighters’ F&V consumption.

## 2. Materials and Methods

### 2.1. Study Design

This was a feasibility study with a cluster-randomized controlled trial design used to investigate the application of a healthy eating promotion program delivered to firefighters via WhatsApp. The data were collected by using self-administered questionnaires at the baseline (T_0_) and 3 months after the completion of an 8-week intervention (T_1_). Qualitative surveys were also conducted at T_1_. The questionnaire included: (1) aspects of personal information; (2) working characteristics; and (3) eating habits regarding F&V consumption. In addition, the firefighters’ feedback on this feasibility study was assessed by four open-ended questions. This study was approved by the Hong Kong Polytechnic University (HSEARS20180527001).

### 2.2. Sample Size

For the sample size estimation of a feasibility study such as this, no clear definitions or guidelines have been found [39]. A sample size of 10–15 in a group was probably sufficient [40] and allowed for estimation of the feasibility proportions of adherence and a retention rate within at least ±17% [41,42] using a 95% confidence interval with a power of 80% to detect an effect size of 0.5 [43].

### 2.3. Participants

Male firefighters were recruited by convenience and snowball sampling methods between September 2018 and May 2019. The inclusion criteria included: (1) aged 18 years or older; (2) currently working as firefighters; (3) working on “24 h on and 48 h off” shifts; and (4) owning smartphones with internet access. Firefighters were excluded if they were participating in any other relevant health promotion programs at the time of this study. Three participants dropped out from the study for various personal reasons. Therefore, their data were excluded from the data analysis process.

### 2.4. Procedures

Forty-eight eligible firefighters completed the written consent and questionnaire at T_0_. Then, based on a computerized random-number generator, 23 firefighters from 17 fire stations were allocated to the intervention group (health promotion pamphlet and education materials through WhatsApp) while 25 from six fire stations were allocated to the control group (health promotion pamphlet). Randomization was performed by an independent staff member, who was not involved in the study. All participants were informed that they would receive the health promotion material. However, they were not told the type of health promotion material (i.e., pamphlets and/or WhatsApp) that they would be receiving. Thus, all the participants were blinded in this study.

### 2.5. Interventions

Two parallel arms of the 8-week intervention (health promotion pamphlet and teaching materials through WhatsApp) versus an 8-week control (health promotion pamphlet) were used in this study.

#### 2.5.1. Intervention and Control Groups

**Figure 1:**
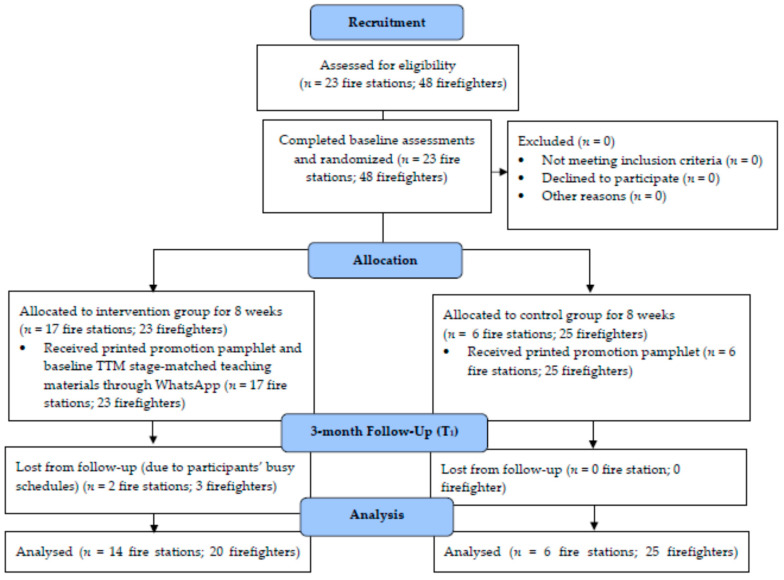
CONSORT diagram.

Both the intervention and control groups received the printed pamphlet at the beginning of the study. In addition, the intervention group also received baseline TTM stage-matched teaching materials via WhatsApp. The number of these materials received depended on the participants’ TTM stages. For instance, those who had been identified initially as being in the precontemplation-stage received pre-contemplation-, contemplation-, preparation- and action-stage teaching material over the 8 weeks at 1–2 week intervals [44] (Figure 1). The pamphlet consisted of teaching material for all four stages. The teaching materials were developed based on a review of literature such as Promoting Healthy Lifestyles: Alternative Models’ Effects (PHLAME) [45], the Centre for Food Safety [46], the Department of Health in HK [47], and the application of TTM for promoting healthy eating [48]. The content of the teaching materials consisted of: (1) a rationale for healthy eating; (2) advantages of consuming F&V; (3) introducing different methods of cooking vegetables; and (4) practical tips for getting enough F&V when eating out or during festival seasons.

#### 2.5.2. Fidelity of Teaching Materials and Pamphlet

A panel of six experts, including three experienced registered nurses, two nutritionists and one dietitian, who all had more than ten years of experience in their respective areas of expertise, were invited to validate the pamphlet and teaching materials with stage-based TTM given via WhatsApp. Each expert completed a checklist, which consisted of all items and features of the intervention that the participants received, from pre-contemplation to action stages, and they were asked to rate each item and feature on a 4-point Likert scale (1 = Not relevant, 2 = Somewhat relevant, 3 = Quite relevant, 4 = Highly relevant). One of the six experts disagreed with others on one item. After adding the wordings “fruit and vegetables” to this item to record weekly healthy eating behavior, all the items were rated either “Quite relevant” or “Highly relevant”.

#### 2.5.3. Validity and Reliability for Questionnaire

The original English of the questionnaire was translated to the Chinese version and two independent professional translators performed the back and forward translations. A content validity index (CVI) of no less than 0.8 was attained from this process. The Chinese version of the questionnaire was examined, using CVI, by a panel of six experts including three experienced registered nurses, two nutritionists, and one dietitian. The questionnaire was considered valid as its CVI was 0.966, with average item CVIs ranging from 0.667 to 1.000 and individual panel members’ CVIs ranging from 0.885 to 0.987. The validated questionnaire was then examined for reliability by having ten firefighters, who met the inclusion criteria for the study, complete it twice with a two-week interval. The concurrence coefficients for these data ranged from 0.704 to 1.000, with a mean of 0.75.

### 2.6. Measures

The study questionnaire consisted of participants’ demographic characteristics (age, marital status, years of experience, and current position), and information on their eating habits including current F&V consumption. However, other daily habits such as drinking alcohol and smoking were not included in this study.

#### 2.6.1. F&V Consumption

The criteria used to measure eating habits, i.e., F&V consumption, were developed based on eating habit studies [17,49,50,51]. These five items were: (1) “On average, how many days do you eat fruits within a week?”; (2) “On the day(s) that you have eaten fruit, how many pieces of fruit do you eat?” (1 = Less than 1, 2 = 1 to 2, 3 = More than 2); (3) “On average, how many days do you eat vegetables within a week?”; (4) “On the day(s) that you have eaten vegetables, how many bowls of vegetables do you eat?” (1 = Less than 1, 2 = 1 to 2, 3 = More than 2); and (5) “How many servings of fruits/vegetables does the respondent usually consume each day?” (1 = Less than 2 servings for fruit and less than 3 servings for vegetables, 2 = ≥2 servings for fruit or ≥3 servings for vegetables).

#### 2.6.2. Practicality and Implementation

Four items were used to evaluate the practicality and implementation of the intervention, including (1) the ease of understanding of the teaching material content and/or pamphlets; (2) the participants’ views of the usefulness of these materials; (3) the data collection venues; (4) the delivery mode of the intervention. Each participant was asked those four items using the same wording and in the same order. The questions were: (1) “What do you think about the content of the teaching materials and/or pamphlets in terms of ease of understanding?”; (2) “What do you think about the content of the teaching materials and/or pamphlets in terms of their usefulness?”; (3) “What do you think about the appropriateness of the location of the venue?” and (4) “What do you think about the delivery mode of the information?”.

### 2.7. Statistical Analysis

All the statistical analyses were conducted using IBM SPSS Statistics for Windows, version 26.0, Armonk, NY: IBM Corp. In order to minimize any bias occurring during the data analysis process, the statistician did not know the group allocations. The participants’ demographic characteristics were presented using descriptive statistics including means and standard deviations (SD) for continuous variables such as age and years of work experience. For discrete variables, such as marital status and current position / major duty, frequencies and percentages were used. The Mann–Whitney U test was used to compare F&V consumption between the control and intervention groups at two single time points (T_0_ and T_1_). Similarly, the Wilcoxon sign-ranked test, a within group test method, was used to test F&V consumption between T_0_ and T_1,_ in the two groups. A *p*-value of less than 0.05 was considered as statistically significant for all tests.

## 3. Results

Of the forty-eight male firefighters recruited, three from the intervention group did not complete the study, hence the response rate was 93.8%. The majority of the participants (*n* = 42, 93%) were frontline firefighters, who are responsible for many physically demanding tasks, including firefighting, emergency, and rescue work. Among the forty firefighters, 28 (62%), 7 (16%), and 5 (11%) were firemen, senior firemen and principal firemen, respectively. There were no significant differences between the intervention and control participants’ demographic characteristics at baseline. The baseline demographic characteristics of the 45 participants are shown in Table 1.

### 3.1. Eating Habits

#### 3.1.1. Number of Days of F&V Intake within a Week

**Figure 2:**
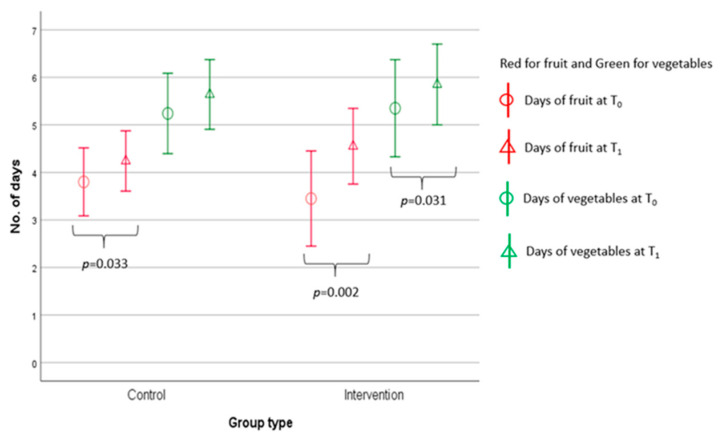
Within-group comparison of number of day(s) of two groups’ F&V consumption between T_0_ and T_1_ by Wilcoxon sign-ranked test.

Both groups had similar patterns in the numbers of days of F&V consumption, with increasing trends (Figure 2). For within-group comparisons, a significant (*p* = 0.033) difference was found in the control group for fruit consumption only, i.e., increased from 3.8 ± 1.7 days to 4.2 ± 1.5 days from T_0_ to T_1_. However, in the intervention group, significant differences were not only found for fruit consumption from 3.5 ± 2.1 days to 4.6 ± 1.7 days (*p* = 0.002); but also for vegetables consumption from 5.4 ± 2.2 days to 5.9 ± 1.8 days (*p* = 0.031). No significant between-group differences were found. Effect size (Cohen’s *d*) of the changes of F&V consumption in the intervention group ranged from small to large (fruit: *d* = 0.6, vegetables: *d* = 0.3). For control group, medium effect size (*d* = 0.3) for fruit and small effect size for vegetables (*d* = 0.2).

#### 3.1.2. Number of Servings of F&V Intake Per Day

The proportions of participants having F&V consumption per day “≥2 servings of fruit or ≥3 servings of vegetables” increased for both groups (Table 2), but the increases were not statistically significant for either within-group or between-group comparisons.

### 3.2. Recruitment

A total of 48 firefighters showed interest and completed the questionnaire at baseline, T_0_. Three firefighters (dropout rate of 6.3%) in the intervention group withdrew from the study after T_0_ because of their excessively busy work and family commitments. Two of them mentioned fully packed working schedules and difficulty in completing the remaining measurement sessions at T_1_. No firefighter reported suffering any adverse effects. There were no missing data from any of the 45 firefighters in this study.

### 3.3. Retention

Forty-five out of 48 firefighters have completed the health promotion program and data collection at T_0_ and T_1_, with a retention rate of 93.8.

### 3.4. Practicality

Forty-eight participants expressed that the contents of the teaching materials and/or pamphlets were easy to follow, read, and understand. The majority of participants (98%) reflected that the information in the teaching materials and/or pamphlets was useful. All participants said that the information in the teaching materials and/or pamphlets could facilitate their food selection and increased their intention to eat two servings of fruits and three servings of vegetables per day.

### 3.5. Implementation

All participants in the intervention group rated the adoption of WhatsApp as the delivery channel of intervention as satisfactory, since they could read the teaching materials at any time and place. All participants in the control group reported that they only went through the printed pamphlets at the beginning of the study. They explained that it was inconvenient to carry the pamphlet from place to place. Two participants in the intervention group were too busy with their work and family commitments and did not complete the study, however their withdrawal was not related to the actual implementation of the intervention.

## 4. Discussion

To the best of our knowledge, our study is the first to have used WhatsApp to promote healthy eating habits in firefighters. The results are promising with regard to retention, practicality, implementation, and increasing F&V intake in the intervention group.

The overall attrition rate of our study was 6.3% (13.0% in intervention group; 0% in control group), which is less than other studies with a typical rate of 17% [40]. The low attrition rate could be due to the WhatsApp reminder messages sent to each participant two days before the data collection [44]. Furthermore, more than ten data collection time slots were arranged to fit in with the participants’ availability, irrespective of public holidays or evenings. The practicality and implementation of the program were supported by the high levels of participants’ satisfaction; they rated the intervention as useful and easy to follow, read, and understand. However, the intervention group had a slightly higher attrition rate than the control group. Attrition can pose a threat to internal and external validity, and reduce statistical power [52]. Thus, one of the purposes of this feasibility study was to identify the types of participants who might tend to drop out, so that appropriate strategies can be implemented in future studies to minimize attrition [52]. A review of our data found that the firefighters in the intervention group were relatively younger and not married. A possible explanation is that younger participants might not have committed strongly to the study [52] due to their over-confidence [53]. This finding is consistent with that of another study, which indicated that single and younger participants were more likely to drop out from an intervention [54]. In addition, those who dropped out did believe at the outset that they had adequate knowledge about healthy eating. They thought their daily vegetable consumption was sufficient, although this was not reflected in the numbers of servings they recorded eating per day; which was less than 1–2 servings. Some studies have found that participants might quit a study if they think they have already gained what they needed from the intervention [55,56]. Based on our study results, the effect size would be 0.25; thus, the sample size for the main study would be 446, with 223 in each group. Strategies to increase the commitment of the young and single firefighters involved in the main study are essential. Heiman, & Olenik-Shemesh (2019) found that younger men were not satisfied with their body appearance, particularly having a belly. In future studies, improving their body image and increasing their awareness of the body could be reinforced as the advantages of increasing consumption of F&V among younger firefighters [57].

The high retention rate of 100% in the control group may have occurred because the data collection from 76% of the firefighters was conducted at their worksites, while none of the participants in the intervention group completed the data collection process at their worksites. To improve the retention rate, attention should be paid to the single and young firefighters, such as providing them with more support and key messages in the beginning of the intervention. Additionally, support from senior management is essential. Several studies have shown that support from senior management contributed to the effectiveness of worksite healthy eating promotion programs [58,59]. This suggests that it will be necessary to introduce our successful WhatsApp-delivered intervention to senior management to increase their support for firefighters to participate in the study.

Our study also found that the intervention group had significantly improved the number of days taking F&V (Figure 2); and had greater percentages of servings of F&V (Table 2) than those in the control group. One of the possible explanations is that the WhatsApp teaching material was TTM stage-matched [60]. Various studies have identified that the TTM stage-matched intervention can increase the intakes of F&V [60,61], because it is a sequential behavioral change model based on the participants’ readiness to adopt healthy lifestyle behaviors [36]. The strength of the TTM is that it treats behavioral change as dynamic rather than an “all or nothing” phenomenon [61]. The TTM consists of a five-stage continuum: people (1) in the precontemplation stage do not have any intention to take action to change their behaviors in the subsequent six months; (2) in the contemplation stage do intend to change their behaviors in the next six months; (3) in the preparation stage are intending to take action to change their behaviors in the next 30 days; (4) in the action stage have successfully changed their behaviors but for less than six months; and (5) in the maintenance stage have sustained their behavioral change successfully for six months or more [61]. Thus, our stage-matched intervention was a relevant, stage-specific, factors-orientated intervention designed especially for a particular stage of change [62] and to motivate behavioral changes.

It is worth noting that, although the intervention group had shown more behavioral change in F&V consumption, the control group also showed improvement. The effect of theory-driven design of the teaching materials, as mentioned, might explain these results. The printed pamphlets given to both the intervention and control groups were TTM-based. In addition to the TTM-based printed pamphlet, the teaching material received by the intervention group through WhatsApp was TTM stage-matched. We also found that the comparable results between groups (i.e., the non-significant differences in the numbers of F&V servings) were consistent with other studies measured at 2 months [63] and 3 months [64] follow-up. Other studies have found increases in numbers of either fruit or vegetable servings consumed up to 6 months after an intervention [65]. This suggests that it might take 6 months or even 1 year to observe the effects of eating habit changes. Thus, the duration of this intervention study should be extended, for example to 6 months or even 1 year after the intervention, in order to assess the follow-up effect of the study.

Furthermore, the success of the intervention can be attributed to the delivery method. Shahril and colleagues [66] found that printed pamphlets are easy to access for repeated reading. However, in the current digital world, where printed material might be outdated, WhatsApp might be even easier to use with quicker access at any time in various locations [67]. In our evaluative qualitative surveys, the participants also indicated that they could access the WhatsApp teaching material any time, but not the pamphlet. Due to the participants’ mobile work environment, our study results support WhatsApp as a promising alternative tool to promote healthy eating habits [68].

### Strengths and Limitations

The main strengths of our study are twofold. First, the sample size of 45 was adequate for a feasibility study, as this allowed more than 15 per group [31]. Second, the participants and statistician were blind to the intervention. However, our study had some limitations. The uneven distribution of cluster sizes, ranging from 1 to 19, might have affected the effects of the intervention. As mentioned, there is a need to improve this uneven distribution in a future main study, by securing senior management support. The researcher collected data should also be blinded in a future study.

## 5. Conclusions

The results of this study demonstrate the baseline TTM stage-matched teaching materials delivered via WhatsApp and TTM-based pamphlets were feasible for promoting healthy F&V eating habits in firefighters. The intervention group showed a greater improvement in F&V consumption than the control group. Furthermore, the study results support the feasibility of the methods of recruitment, retention, practicality, and implementation used. Longer follow-up studies are needed to identify the most effective TTM-based approach for increasing F&V consumption. Additionally, the support from senior management is important since this will allow other firefighters to participate, and future results can thus be generalized.

## Figures and Tables

**Table 1:** Baseline demographic characteristics of the participants (N = 45).

Characteristics	Total (N = 45)	Intervention (*n* = 20)	Control (*n* = 25)
Age (Years)	35.0 ± 9.6	32.9 ± 9.5	36.6 ± 9.6
Years of work experience (Years)	11.3 ± 9.9	9.4 ± 9.6	12.8 ± 10.1
Marital status (%)			
Single	47	55	40
Married	51	40	60
Divorced	2	5	0
Current major duty (%)			
Front-line	93	95	92
Management work	4	0	8
Others	2	5	0
Current position (%)			
Fireman	62	70	56
Senior fireman	16	5	24
Principal fireman	11	10	12
Probationary station officer	9	15	4
Senior station officer	2	0	4

**Table 2:** Between-group comparison of the numbers of servings of F&V intake per day between groups at each time point (Mann–Whitney U test).

Time	Serving	Total (N = 45) n (%)	Intervention (*n* = 20) n (%)	Control (*n* = 25) n (%)	*p*
T_0_	<2 servings of fruits and <3 servings of vegetables ^1^	37 (82)	17 (85)	37 (82)	0.304
	≥2 servings of fruits or ≥3 servings of vegetables	8 (18)	3 (15)	8 (18)	
T_1_	<2 servings of fruits and <3 servings of vegetables	28 (62)	12 (60)	28 (62)	0.789
	≥2 servings of fruits or ≥3 servings of vegetables	17 (38)	8 (40)	17 (38)	

^1^ <2 servings of fruits and <3 servings of vegetables: less than recommended for both F&V.

## Data Availability

The data presented in this study are available on reasonable request from the corresponding author.
